# Thiazolidinediones: the Forgotten Diabetes Medications

**DOI:** 10.1007/s11892-019-1270-y

**Published:** 2019-11-27

**Authors:** Harold E. Lebovitz

**Affiliations:** SUNY Downstate Medical Center, 450 Clarkson Avenue, Box 1205, Brooklyn, NY 11203 USA

**Keywords:** Type 2 diabetes, PPARγ agonists, Insulin resistance, CV outcomes, Heart failure, Bone fractures

## Abstract

**Purpose of Review:**

Thiazolidinediones (TZDs) are the only pharmacologic agents that specifically treat insulin resistance. The beneficial effects of TZDs on the cardiovascular risk factors associated with insulin resistance have been well documented. TZD use has been limited because of concern about safety issues and side effects.

**Recent Findings:**

Recent studies indicate that cardiovascular toxicity with rosiglitazone and increase in bladder cancer with pioglitazone are no longer significant issues. There are new data which show that pioglitazone treatment reduces myocardial infarctions and ischemic strokes. New data concerning TZD-mediated edema, congestive heart failure, and bone fractures improves the clinician’s ability to select patients that will have minimal significant side effects.

**Summary:**

Thiazolidinediones are now generic and less costly than pharmaceutical company–promoted therapies. Better understanding of the side effects coupled with clear benefits on the components of the insulin resistance syndrome should promote TZD use in treating patients with type 2 diabetes.

## Introduction

For years there was an intense debate as to whether the primary defect in type 2 diabetes (T2D) was insulin resistance or insulin secretory deficiency. This conflict has been resolved in that hyperglycemia only occurs when insulin secretion is insufficient to overcome barriers to insulin action [1•]. Thus, when insulin action is normal, hyperglycemia occurs when absolute insulin secretion is deficient. In contrast, when insulin action is impaired (insulin resistance), hyperglycemia occurs when insulin secretion is inadequate to overcome the insulin resistance. While a few investigators suggest that hyperinsulinemia itself may be a primary cause of insulin resistance, the overwhelming data indicate that insulin resistance is a primary defect due to abnormalities in the distribution of adipose tissue products in hepatic and extrahepatic tissues [2••, 3••, 4]. These ectopic increases in lipids define the metabolic syndrome. The metabolic syndrome results in increases in atherosclerotic diseases, hepatic steatosis and steato-hepatitis, and hypertension [5, 6••]. Insulin resistance increases the prevalence of T2D sixfold as marginal insulin secretion becomes inadequate insulin secretion [7, 8].

Patients with T2D may have hyperglycemia and no metabolic syndrome (up to 15% of patients with T2D) or hyperglycemia with the metabolic syndrome (85% of patients with T2D) [9, 10••]. Data from NHANES III participants 50 years or older showed that patients with T2D and no metabolic syndrome had a prevalence of coronary heart disease of 7.5% which did not differ from a non-diabetic population with no metabolic syndrome (prevalence 8.7%) [10••]. Patients with no diabetes and the metabolic syndrome had a 13.9% prevalence of coronary heart disease and those with diabetes and the metabolic syndrome had a 19.2% prevalence of coronary heart disease [10••]. These and other data suggest that the metabolic syndrome is the driving factor in the development of cardiovascular disease and that hyperglycemia is a factor that exaggerates cardiovascular disease in the metabolic syndrome setting.

**Fig. 1:**
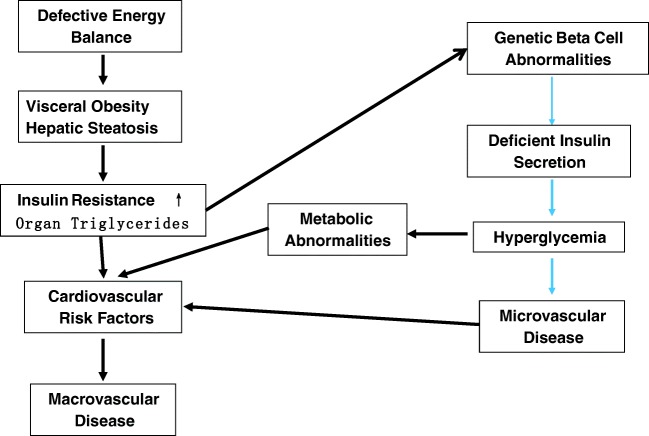
Insulin-resistant type 2 diabetes is the co-existence of two separate metabolic abnormalities: a primary beta-cell disease: diabetes mellitus and a lipid distribution abnormality which creates insulin resistance, the metabolic syndrome, and increased cardiovascular disease. These abnormalities can be separate or co-exist. These abnormalities can interact in susceptible populations leading to an increase in the prevalence of diabetes and an increase in atherosclerotic disease

This formulation of insulin-resistant T2D as two interacting pathophysiologic entities (Fig. 1) suggests that appropriate treatment should target both the insulin resistance and the inadequate insulin secretion. Therapeutic agents such as insulin, sulfonylureas, and meglitinides target primarily insulin availability [11]. Glucagon-like peptide 1 (GLP-1) receptor agonists and dipeptidyl dipeptidase 4 (DDP-4) inhibitors increase insulin secretion and decrease hyperglucagonemia, thereby lowering hyperglycemia [12–14]. GLP-1 receptor agonists have modest effects in decreasing body weight and lowering systolic blood pressure [12, 13]. The sodium-glucose transport 2 (SGLT-2) inhibitors lower blood glucose through an increase in renal excretion of glucose with a secondary benefit of decreasing glucose toxicity [15]. They increase hepatic glucose production, glucagon secretion, ketogenesis, and lipid oxidation [15, 16]. A significant side effect of their treatment is a modest weight loss, a decrease in blood pressure, and a modest reduction in insulin resistance [15, 16]. Weight loss itself decreases hepatic triglycerides, peripheral and visceral adipose tissue mass, and plasma triglycerides [17•]. GLP-1 receptor agonists and SGLT-2 inhibitors secondarily decrease insulin resistance in patients in proportion to their effect in promoting weight loss. Thiazolidinediones (TZDs) decrease insulin resistance directly through activation of PPARγ receptors which facilitate differentiation of mesenchymal stem cells into adipocytes, promote lipogenesis in peripheral adipocytes, decrease hepatic and peripheral triglycerides, decrease activity of visceral adipocytes, and increase adiponectin [18••, 19, 20]. These primary effects of TZDs markedly ameliorate insulin resistance and the metabolic syndrome and decrease insulin requirements [18••, 19, 20]. While metformin decreases hepatic insulin resistance, it has little or no primary effect on peripheral insulin resistance [21, 22]. Metformin’s effects on hepatic insulin resistance are likely to be due to its inhibition of mitochondrial complex 1 resulting in defective cyclic AMP signaling [22].

## Clinical Benefits of Thiazolidinediones

The metabolic consequences of insulin resistance are an increase in the incidence of T2D and an increase in cardiovascular diseases [7, 8]. Insulin resistance decreases pancreatic beta-cell function by causing compensatory hyperinsulinemia, beta-cell lipotoxicity, and increasing islet inflammation [22, 23]. These factors result in increased beta-cell apoptosis and progressive decreases in insulin secretory capacity. Insulin resistance is associated with increases in multiple cardiovascular risk factors [2••, 3••, 4, 5, 6•]. Insulin resistance creates a dyslipidemia consisting of increases in plasma-free fatty acids and triglycerides, decreases in HDL-cholesterol, decreases in adiponectin, and increases in small dense LDL particles [2••, 6•, 18••, 24••]. Insulin resistance is a major cause of hepatic steatosis and steato-hepatitis (NASH) [24••]. Insulin resistance creates a procoagulant state with increases in fibrinogen and plasminogen activator inhibitor-1 (PAI-1) [5, 6•]. Insulin resistance increases inflammation and is associated with an increase in C-reactive protein (CRP), IL-6, and TNFα [25, 26]. Insulin resistance is associated with endothelial dysfunction as measured by decreased vasodilatation, increase in adhesion molecules, and an increase in cellular proliferation [5, 27]. An increase in hepatic and visceral fat leads to ectopic deposits of lipids in tissues such as skeletal muscle, myocardial muscle, and endothelial cells, causing a decrease response to insulin [2••, 3••, 24••]. These ectopic deposits of intracellular lipids within muscle and endothelial cells increase serine and threonine phosphorylation and decrease tyrosine phosphorylation of insulin receptor substrates and phospho-inositol 3-kinase, thereby decreasing intracellular insulin signaling [2••, 24••]. An increase in beta-cell lipids decreases its insulin secretory function [23, 28].

Significant weight loss in obese insulin-resistant individuals will secondarily reduce insulin resistance and the components of the insulin resistance syndrome [17, 29]. TZDs through their activation of PPARγ have primary effects on adipose tissue and decrease insulin resistance by reducing hepatic triglycerides, decreasing visceral fat mass and activity and increasing subcutaneous fat mass [2••, 18••, 19, 20]. This primary action of TZDs in reducing insulin resistance accounts for their beneficial effects in ameliorating the detrimental effects of insulin resistance. Table 1 describes the beneficial effects of TZDs in the treatment of patients with T2D [2••, 18••, 20, 30, 33]. TZDs decrease both fasting and postprandial hyperglycemia by decreasing insulin resistance and allowing endogenous insulin to be more effective. Where measured, TZDs have a more durable effect in reducing hyperglycemia than other antihyperglycemic agents [34]. The multiple effects in decreasing dyslipidemia, endothelial dysfunction, inflammation, and the procoagulant state and increasing adiponectin are thought to provide some potential cardiovascular benefits. The effects of TZDs in reducing hepatic steatosis suggest potential therapeutic benefits in reducing NASH. The reduction in insulin resistance in patients with prediabetes decreases their rate of progression to T2D, presumably by preserving beta-cell function [31•, 32•]. Between 2000 and 2008, pioglitazone and rosiglitazone were among the most widely prescribed antidiabetic medications. However, there were increasing concerns about TZDs’ side effects: increased fluid retention; increased incidence of heart failure; weight gain; and increased peripheral fractures [35•, 36••]. Regulatory concerns about cardiovascular safety issues with rosiglitazone and increases in bladder cancer with pioglitazone markedly reduced the availability of these drugs [37–39]. Since the regulatory issues appear to have been greatly exaggerated and TZDs are the only agents that primarily target insulin resistance, the use of these relatively inexpensive drugs justifies a re-evaluation of their clinical use.

**Table 1:** Beneficial effects of thiazolidinediones in the treatment of patients with insulin resistance [3••, 18••, 20, 25–27, 30, 31•, 32•]

1. Improves insulin sensitivity in insulin-resistant individuals from 25 to 68% depending on the study and technique used (euglycemic-hyperinsulinemic clamp, Bergman minimal model, Homeostasis Model Assessment of Insulin Resistance (HOMA-IR))	
2. Effects on adipose tissue a. Increase subcutaneous adipose tissue mass approximately 3.5% b. Little no effect on visceral adipose tissue mass	
3. Effects on dyslipidemia a. Decreases plasma-free fatty acids 25 to 35% b. Increase in plasma HDL cholesterol 10 to 20% c. Plasma triglycerides reduced particularly if baseline value > 200 mg/dL d. Small dense LDL particles are converted to large buoyant LDL particles e. Increases adiponectin	
4. Decrease in hepatic triglyceride concentration (hepatic steatosis)	
5. Effects on endothelium a. Vasodilatation is increased b. Systolic and diastolic blood pressure decreased 4 to 5 mmHg c. Production of adhesion molecules (VCAM 1, ICAM 1) are decreased d. Vascular smooth muscle cell proliferation is inhibited e. Neointimal tissue proliferation after coronary stent implantation in type 2 diabetes is reduced 50–70%	
6. Effects on inflammation a. Reduces mean plasma C-reactive protein (CRP) by 25–30% b. WBC counts and metalloproteinase are reduced	
7. Effects on procoagulant state a. Reduces plasminogen activator inhibitor 1 (PAI-1) approximately 25% b. Reduces plasma fibrinogen	
8. Effect on glycemic control a. Thiazolidinedione reduction in glycated hemoglobin (HbA1c) results from decreases in both fasting and postprandial hyperglycemia b. The magnitude of decrease in HbA1c is a function of the magnitude of improvement in resistance and the extent of residual insulin secretion c. In large clinical trial such as PROactive, HbA1c was reduced − 0.8% from baseline 7.8% with 53% reduction in insulin dose d. The ADOPT study data showed that rosiglitazone had a significantly greater durability in controlling glycemia after 5 years of treatment of patients with type 2 diabetes than either metformin or glyburide	
9. Effect in preserving beta-cell function a. In separate studies, rosiglitazone (DREAM) and pioglitazone (ACT NOW) decreased the progression of prediabetes to diabetes by 60% and 72%, respectively, after a median treatment of 3.0 and 2.4 years b. Decrease in progression from prediabetes to diabetes in both trials was most closely associated with preservation of beta-cell insulin secretory function	

## Effects of Thiazolidinediones on Cardiovascular Outcomes

Since TZD treatment improves insulin resistance and decreases many cardiovascular risk factors, it had been proposed that treatment of patients at high risk for cardiovascular events with TZDs might have a beneficial effect in reducing long-term cardiovascular events. Clinical studies have examined the effects of pioglitazone and rosiglitazone on cardiovascular outcomes, and several meta-analyses and large database analyses have compared their relative cardiovascular outcomes.

### Pioglitazone

The PROactive (PROspective pioglitAzone Clinical Trial In Macrovascular Events) trial randomized 5238 patients with T2D and evidence of macrovascular disease between May 2001 and April 2002 into a controlled, double-blind, cardiovascular outcome trial with pioglitazone titrated 15 to 45 mg/day or placebo [40••]. The primary endpoint was a composite of all-cause mortality, non-fatal myocardial infarction (including silent myocardial infarction), stroke, acute coronary syndrome, endovascular or surgical intervention in coronary or leg arteries, and amputation above the knee. After an average duration of 34.5 months, pioglitazone failed to meet the primary endpoint of a reduction in at least one those composite hard or soft cardiovascular endpoints (hazard ratio (HR) 0.90, *p* = 0.095). There was a significant reduction in the main secondary endpoint of all-cause mortality, non-fatal myocardial infarction (excluding silent myocardial infarctions), and stroke (HR = 0.84, *p* = 0.027) [40••]. Pioglitazone caused a significant increase in hospitalization for heart failure (149 vs. 108 placebo-treated patients, *p* = 0.007); however, mortality rates for heart failure did not differ from the placebo group. The results of the PROactive study were extensively debated because of the inclusion of so many diverse components in the primary endpoint, the exclusion of silent myocardial infarctions in some analyses, and differences in numbers of events in the primary and secondary analyses. Some reviewers claimed that the results showed a significant decrease in cardiovascular outcomes, and others argued that the design and statistical analyses were flawed, and the conclusions reached by the investigators were invalid. One finding that everybody agreed upon was that pioglitazone increased the incidence of hospitalizations but not the mortality for congestive heart failure. Weight gain (pioglitazone 3.6 kg vs. placebo − 0.4 kg) and peripheral edema (pioglitazone 562 patients vs. placebo 341 patients) were increased in the pioglitazone-treated population as was expected from its known side effects [40••].

While the data do not prove conclusively that pioglitazone reduces cardiovascular events, the data are consistent with a benefit. The weakness in the PROactive study lies in the endpoint assumptions made. It should be noted that this was the first major cardiovascular outcome study evaluating a single antidiabetic treatment.

The results of two recent, large, well-designed trials have provided new meaningful data on the effects of pioglitazone on cardiovascular outcomes. The Insulin Resistance Intervention after Stroke (IRIS) trial, which was a multicenter, double-blind trial that investigated the effect of pioglitazone on future cardiovascular events in patients who had insulin resistance and had had a recent ischemic stroke or a transient ischemic attack [41••]. Three thousand eight hundred seventy-six participants without diabetes were treated with pioglitazone 45 mg or a placebo. Insulin resistance was defined as a measured Homeostasis Model Assessment, HOMA-IR > 3.0. The primary outcome was fatal or non-fatal stroke or myocardial infarction. By 4.8 years, 175 of 1939 (9.0%) patients treated with pioglitazone and 228 of 1937 (11.8%) placebo-treated patients had a primary event. The HR for pioglitazone treatment was 0.76 (95% CI 0.62–0.93). Diabetes developed in 73 participants (3.8%) receiving pioglitazone and 149 participants (7.7%) receiving placebo, HR = 0.48, *p* = 0.001. No significant difference occurred in all-cause mortality (HR = 0.93). Pioglitazone therapy was associated with an increase in weight gain > 4.5 kg (52.2% vs. 33.7%), *p* < 0.001; edema 35.6% vs. 24.9%, *p* < 0.001; and bone fractures requiring surgery or hospitalization 5.1% vs. 3.2%, *p* = 0.003 [41••].

The TOSCA-IT study was a large multicenter trial which evaluated the cardiovascular outcomes of the long-term effect of adding pioglitazone vs. a sulfonylurea to patients with T2D inadequately controlled on monotherapy with metformin [42]. From September 18, 2008, to January 15, 2014, 3028 patients inadequately controlled with metformin were randomized to add either pioglitazone (15 to 45 mg) or a sulfonylurea (5–15 mg glibenclamide, 2–6 mg glimepiride, or 30–120 mg gliclazide). The study was unblinded, but adjudicators were blinded. Primary outcome was a composite of all-cause mortality, non-fatal myocardial infarction, non-fatal stroke, or urgent coronary revascularization. Of the 1493 participants randomized to sulfonylureas, 24 were taking glibenclamide, 723 glimepiride, and 745 gliclazide. At baseline, 335 (11%) had had a previous cardiovascular event. The study was stopped at a mean follow-up of 57.3 months because of the lack of any difference in the primary endpoint (pioglitazone 105 participants, 1.5 events/100 person-years; sulfonylureas 108 participants, 1.5 events/100 person-years, HR = 0.96, *p* = 0.79). Hypoglycemia occurred in 148 (10%) participants in the pioglitazone group and in 508 (34%) participants in the sulfonylurea group. Weight gain < 2 kg on average occurred in both groups. Rates of heart failure, bladder cancer, and fractures were not significantly different between groups [42]. It is of interest that the results of TOSCA-IT and RECORD are very similar, and raise the possibility that the results of cardiovascular outcome studies may differ depending on whether the comparator is placebo or an active antihyperglycemic agent [42, 43•].

The effects of pioglitazone in patients with previous strokes or transient ischemic attacks has been further investigated by subanalysis of previous clinical trials or meta-analyses of several studies. In the PROACTIVE study, a subanalysis was done of the 486 pioglitazone-treated patients and the 498 placebo-treated patients that had had a previous stroke [44]. Pioglitazone reduced fatal and non-fatal stroke 5.6% vs. placebo 10.2%, *p* = 0.0085; HR 0.53, and the composite cardiovascular endpoint of cardiovascular death, non-fatal myocardial infarction, and non-fatal stroke 13.0% vs. placebo 17.7%, *p* = 0.0467; HR = 0.72 [44]. In a secondary analysis of the IRIS trial, patients were stratified above and below the median for risk for stroke and myocardial infarction at baseline [45]. The efficacy of pioglitazone for preventing stroke or myocardial infarction and for safety (death, heart failure, weight gain, and fracture) was determined for each stratum. In the low-risk stratum, the risk for pioglitazone-treated participants was 6.0% and for placebo-treated participants 7.9%. The absolute difference was − 1.9% (95% CI = −4.4 to 0.6%). Among higher risk patients, pioglitazone 14.7% placebo 19.6% with an absolute difference of − 4.9% (95% CI = −8.6 to 1.2), HR = 0.77 vs. 0.75 [45]. Pioglitazone increased weight more in higher risk patients. Fracture risk was greatest in the high-risk group [45].

Meta-analysis of 3 studies with 4980 participants showed that pioglitazone treatment in participants with insulin resistance, prediabetes, and diabetes had a lower risk of recurrent stroke, HR = 0.68, *p* = 0.01, and of future major vascular events, HR = 0.75, *p* = 0.0001. There was no evidence of an effect on all-cause mortality, and heart failure [46].

Many small trials have investigated the effects of pioglitazone on cardiovascular endpoints or their surrogates. These studies by themselves are not powered enough or of long enough duration to provide significant information about clinical cardiovascular outcomes. They have been included in systemic reviews and meta-analyses. However, the analyses in these reviews and meta-analyses are dominated by the data from the 9114 subjects from the PROactive and IRIS studies and therefore provide little additional information. An exception is the PERISCOPE study which measured coronary atheroma volume by intravascular ultrasonography in a double-blind, randomized trial of pioglitazone 15 to 45 mg vs. glimepiride 1 to 4 mg in participants with type 2 diabetes [47]. After 18 months, percent atheroma volume decreased 0.16% in the pioglitazone-treated participants and increased 0.73% in the glimepiride-treated participants (*p* = 0.002) [47]. These data along with several other studies showing that pioglitazone decreased coronary restenosis after a stent placement indicate that pioglitazone decreases the rate of coronary atherosclerosis.

### Rosiglitazone

Several early clinical trials involving rosiglitazone treatment of participants with prediabetes or T2D showed an imbalance of more cardiovascular events in the rosiglitazone arm compared with placebo. In 2000, the European Regulatory Agency requested a post-marketing study of long-term cardiovascular morbidity and mortality in participants with T2D treated with rosiglitazone (RECORD study). In 2007, Nissen and Wolski published a meta-analysis of 42 clinical trials which showed that rosiglitazone increased myocardial infarction (odds ratio (OR) = 1.43, 95% CI = 1.03–1.98, *p* = 0.03) and death from cardiovascular causes (OR = 1.64, 95% CI = 0.98–2.74, *p* = 0.060) compared with the control group [37]. Another meta-analysis published the same year involving 14,291 participants from 4 clinical trials reported that rosiglitazone increased myocardial infarctions (RR = 1.42, 95% CI = 1.06–1.93, *p* = 0.02) and heart failure (RR = 2.09, 95% CI = 1.52–2.88, *p* < 0.001) compared with the control groups [38]. No increased risk of cardiovascular mortality was found (RR = 0.90, *p* = 0.53) [38].These publications and the results of several other meta-analyses and database analyses served to raise significant safety concerns about rosiglitazone, and the US FDA assembled several advisory board meetings to review rosiglitazone safety data and make recommendations. An advisory board in July 2007 voted 20:3 that the evidence indicated that rosiglitazone increased the risk of cardiovascular events and 22:1 that the overall risk benefit ratio justified its continuing marketing [48, 49]. Despite the publication of RECORD which showed no differences in cardiovascular events or death from rosiglitazone treatment compared with metformin or sulfonylurea treatments, the European Marketing Authority recommended removal of rosiglitazone from the European market on September 23, 2010 [50, 51•, 52•].

A retrospective analysis comparing rosiglitazone to pioglitazone in 2010 concluded that rosiglitazone was associated with an increased risk of composite cardiovascular events (acute myocardial infarction, stroke, heart failure, or death) (HR = 1.18, 95% CI = 1.12–1.23) compared with pioglitazone in patients 65 years or older [53]. In 2011, under continuing pressure, the US FDA placed very stringent requirements for the use of rosiglitazone in the USA which virtually abolished its use (10,000 US patients used it in 2012) [54]. In June 2013, another FDA panel reviewed all available data including re-adjudicated RECORD trial data and found no evidence of increased cardiovascular risk with Avandia (rosiglitazone) and voted to remove the restrictions on Avandia marketing in the USA [55]. The FDA removed the restrictions in November 2013, but rosiglitazone use had virtually stopped.

RECORD was an exception to most cardiovascular outcome studies in that it was a comparative study of rosiglitazone vs. an active treatment. The design of PROACTIVE and the GLP-1 receptor agonists and SGLT-2 inhibitors cardiovascular outcome studies compared the pharmacologic drug to a placebo control. The FDA and GlaxoSmithKline agreed that a large randomized, controlled trial of rosiglitazone compared with pioglitazone and placebo on major cardiovascular outcomes (MACE) was necessary to definitively determine the effects of rosiglitazone on cardiovascular outcomes. The TZD study design was to randomize 16,000 participants with T2D to rosiglitazone, pioglitazone, or placebo and follow them for non-fatal myocardial infarction, non-fatal stroke, or death due to cardiovascular causes for approximately 5.5 years [56]. The trial was stopped prematurely on July 21, 2010, after 162 days because of the regulatory issues related to rosiglitazone from FDA Advisory Committee recommendations and a US Senate report in May 2010 [56].

Secondary analyses of the BARI-2D trial which evaluated outcomes of bypass angioplasty revascularization in which patients were treated with rosiglitazone for a mean of 4.5 years reported that neither on treatment nor propensity-matched analysis supported an association of rosiglitazone treatment with an increase in major ischemic cardiovascular events [57]. A secondary analysis of the Veterans Affairs Diabetes Trial reported that rosiglitazone use was associated with decreased risk of the primary cardiovascular composite outcome and cardiovascular death [58]. Rosiglitazone did not lead to a higher risk of myocardial infarction.

Several studies were designed to evaluate the effects of rosiglitazone on specific aspects of coronary artery disease. In an 18-month study of 672 subjects with T2D and at least 1 atherosclerotic plaque with luminal narrowing in a coronary artery who were randomized to rosiglitazone or glipizide, there was no significant difference in percent atheroma volume (95% CI = − 1.46–0.17 *p* = 0.12) [59]. In a controlled, randomized study of 83 participants with diabetes who had percutaneous coronary artery stent placement, rosiglitazone significantly reduced restenosis at 6 months compared with the control treatment (17.6 vs. 38.2%, *p* = 0.030) [60]. Baseline and follow-up glucose and lipid levels between the two groups were not different but rosiglitazone treatment reduced high-sensitivity CRP concentration. The effects of rosiglitazone vs. placebo with standard treatment on echocardiographic function and cardiac status of 224 participants with T2D with left ventricular ejection fraction (LVEF) ≤ 45% and New York Heart Association (NYHA) functional class I or II heart failure were evaluated in a randomized study [61]. LVEF was similar between the groups at baseline and after 52 weeks of treatment. Rosiglitazone treatment resulted in better glycemic control (HbA1c − 0.65%), worsening edema and increased congestive heart failure medications.

## Thiazolidinediones and Weight Gain

Weight gain has been associated with both rosiglitazone and pioglitazone treatments [35•, 40••]. The weight gain appears to be dose-related (1 or 1.5 kg at low doses) and is greater during combination therapy with insulin secretagogues (2 to 3 kg) and remarkably so when TZDs are combined with insulin therapy (3.5 to 6 kg). The weight gain is attributable to several factors: increase in subcutaneous fat mass with either no change or a small decrease in visceral fat mass, fluid retention, and positive calorie balance because of improved glycemic control. Though the increase in subcutaneous fat mass is distressing to patients with T2D who are trying to lose weight, the TZD-mediated changes in fat mass distribution is related to the improvement in insulin resistance and glycemic control.

## Thiazolidinediones: Fluid Retention and Edema

During the pre-marketing clinical trials of pioglitazone and rosiglitazone, it was noted that an increase in peripheral edema and the development of congestive heart failure occurred in a few patients. Patients with NYHA functional class III and IV heart failure had been excluded from the clinical studies. Shortly after rosiglitazone and pioglitazone were marketed for clinical use, data appeared which indicated that edema and congestive heart failure were significant complications of TZD treatment of patients with diabetes mellitus. The magnitude of the problem was sufficiently important that the American Diabetes Association and the American Heart Association held a Consensus Conference to discuss and publish a Consensus Statement on “Thiazolidinedione Use, Fluid Retention, and Congestive Heart Failure” [35•].

The consensus derived from meta-analyses and large databases was that monotherapy with either pioglitazone or rosiglitazone is associated with a 3 to 5% incidence of peripheral edema which increases to 7.5 to 8% when they are combined with other antidiabetic agents such as sulfonylureas [35•]. When TZDs are combined with insulin therapy, the incidence of peripheral edema is approximately 15 to 16% as compared with insulin alone which is 5 to 7% [35•].

Initially, it was thought that activation of PPARγ receptors in the renal collecting duct epithelium’s sodium channel (ENaC) was responsible for TZD-related peripheral edema, hemodilution, and even macular edema [62]. It now appears that sodium transporters in the proximal tubule including NHE3 also contribute to sodium reabsorption [63]. Increased vascular permeability through increased vascular endothelial growth factor secretion and decreased systemic vascular resistance are non-kidney factors that contribute to the edema [63].

Table 2 presents data on TZD-mediated edema from randomized, controlled clinical trials and a large French Pharmacovigilance data base. The incidence of TZD-induced edema depends on drug dose, the stage of glucose intolerance, and the magnitude of the underlying cardiovascular disease. The lowest incidence is in prediabetes (DREAM study), with intermediate incidence in T2D (ADOPT and French PharmacoVigilance studies), and the highest in those with advanced cardiovascular disease (PROactive and IRIS studies) [31•, 34, 64••, 65••, 66•]. The extent to which TZD use exacerbates fluid retention in congestive heart failure is unclear [67].

**Table 2:** Thiazolidinediones: edema reported in randomized controlled clinical trials (RCT) and a French PharmacoVigilance Database

Study	Population	Drug	Number	Duration of trial	Edema	Edema without HF	Patients without edema developing HF	Edema before serious HF
PROactive [40••, 64••]	Type 2 DM with previous CV events or high CV risk	Pioglitazone	2065	Mean 34.5 months	713 (27.4%)	563 (21.6%)		51/149 (34.2%)
		Placebo	2663		419 (15.9%)	341 (13.0%)		26/108 (24.1%)
		*p* value			*p* < 0.001	*p* < 0.0001		
RECORD [43••]	Type 2 DM	Rosiglitazone	2220	Mean 5.5 years				
		Sulfonylurea/metformin	2227					
		*p* value						
DREAM [31•]	Patients with IFG and IGT	Rosiglitazone	2365	Median 3 years	439 (4.8%)			
		Placebo	2634		41(1.6%)			
IRIS [41, 65]	Patients after ischemic stroke and with insulin resistance. No diabetes	Pioglitazone	1923	5 years	334/1579 (18%) Adjudicated 12.2%		2.7%	4.7%
		Placebo	1928		228/1700 (12%) Adjudicated 11.0%		2.8%	5.7%
		*p* value						
APPROACH [59]	Type 2 DM with known coronary atherosclerosis	Rosiglitazone	333	Median 18 months	29/333 (9%)			
		Glipizide	339		24/339 (7%)			
		*p* value			*p* = 0.48			
GSK CV function study [35•]	Type 2 DM	Rosiglitazone	104	1 year	7 (6.7%)			
		Glyburide	99		1 (1.0%)			
		*p* value						
Echocardiographic study (Wilding) [61]	Type 2 DM NYHA class I or II heart failure	Rosiglitazone	110	52 weeks	28 (25.5%)			
		Control	114		10 (8.8%)			
		*p* value			*p* = 0.005			
ADOPT [34]	Type 2 DM	Rosiglitazone	1456	Median 4 years	205 (14.1%)			
		Glyburide	1441		123 (8.5%)			
		Metformin	1454		104 (7.2%)			
		*p* value						
French PharmacoVigilance	Type 2 DM	TZD	161	2002–2006	29 (18%)			
		Non-TZD	2134		128 (0.8%)			
		*p* value			*p* < 0.001			

## Thiazolidinediones and Heart Failure

Heart failure is the most significant side effect of treatment with TZDs. Many meta-analyses and database analyses have been published and have concluded that both pioglitazone and rosiglitazone increase the incidence of heart failure in patients with T2D [68–71]. However, they cannot provide data on the severity, progression, and clinical outcome of the patients. Table 3 presents the available data on TZD treatment and the development of heart failure in randomized, controlled clinical trials. Data are available in patients with insulin-resistant and no diabetes, prediabetes, ordinary T2D, and T2D with previous cardiovascular events. Patients with prediabetes treated with TZDs have a very low incidence of heart failure (< 1%) [31•]. Patients with T2D, similarly, had a very low incidence of heart failure (< 1%) [34]. Patients without diabetes but with insulin resistance and a previous ischemic cerebrovascular event had an increase in the incidence of heart failure and hospitalization for heart failure, but these were not related to pioglitazone treatment [65]. VA ambulatory patients with heart failure and diabetes had an increase in heart failure hospitalizations and death over a 2-year follow-up period and they were unrelated to TZD treatment [72••]. Three studies (PROactive, RECORD, and A GSK-sponsored ECHOCARDIOGRAPHIC study) showed significant increases in heart failure hospitalizations in patients treated with a TZD [43••, 61, 64••]. The populations in these three studies had in common a long duration of diabetes and a high prevalence of preceding cardiovascular events. While death from heart failure was increased by rosiglitazone in the RECORD study, death was not increased by pioglitazone in PROactive. It appears that TZDs increase edema and heart failure requiring additional treatment or hospitalization in patients with T2D with significant preceding cardiovascular disease. The available data indicate that pioglitazone treatment does not increase death from heart failure.

**Table 3:** Thiazolidinediones and development of heart failure: randomized, controlled clinical trials in participants with insulin-resistance and either free of diabetes, with prediabetes, or with type 2 diabetes

Study	Population	Drug	Number	Duration of trial	HF occurrence	HF hospitalized	HF mortality
PROactive [64••]	Type 2 DM with previous CV events or high risk	Pioglitazone	2065	Mean 34.5 months	306 (14.6%)	149 (6%)	25(1%)
		Placebo	2663		220 (8.3%)	108 (4%)	22 (1%)
		*p* value			0.003	0.007	0.634
RECORD [43••]	Type 2 DM	Rosiglitazone	2220	Mean 5.5 years	61(2.7%)	57 (2.57%)	4 first event 10 total
		Sulfonylurea/metformin	2227		29 (1.1%)	29 (1.1%)	0 first event 2 total
		*p* value			< 0.001	Not provided	Not provided
DREAM [31•]	Patients with impaired fasting glucose (IFG) and impaired glucose tolerance (IGT)	Rosiglitazone	2365	Median 3 years	14 (0.59%)		
		Placebo	2634		2 (0.07%)		
		*p* value			< 0.01		
IRIS [65]	Patients after ischemic stroke and with insulin resistance. No diabetes	Pioglitazone	1923	5 years	67 (3.5%)	44 (2.3%)	1 (0.0005%)
		Placebo	1928		66 (3.4%)	36 (1.87%)	2 (0.001%)
		*p* value			0.89	0.35	0.51
APPROACH [59]	Type 2 DM with known coronary atherosclerosis	Rosiglitazone	333	Median 18 months	8 (2.4%)		
		Glipizide	339		3 (0.9%)		
		*p* value			0.14		
Echocardiographic study (Wilding) [61]	Type 2 DM NYHA class I or II heart failure	Rosiglitazone	110	52 weeks		36 (32.7%)^a^	
		Control	114			20 (17.5%)^a^	
		*p* value				0.037	
ADOPT [34]	Type 2 DM	Rosiglitazone	1456	Median 4 years	9 (0.6%)		
		Glyburide	1441		4 (0.3%)	1	
		Metformin	1454		8 (0.55%)	1	
		*p* value			Vs. met 0.26		
VA ambulatory [72••]	Ambulatory patients with diabetes mellitus and heart failure	Pioglitazone or rosiglitazone	818	2 years		134 (16.4%)	168 (20.5%)
		No insulin sensitizers	4700			741 (15.8%)	1192 (25.4%)
		*p* value				0.97	0.80

^a^Worsening heart failure requiring increased medications

Rosiglitazone treatment of patients with T2D and NYHA functional class I or II heart failure for 52 weeks did not affect LVEF even though the patients experienced new or worsening edema and increased congestive heart failure medications [61].

## Thiazolidinediones and Bone Fractures

The second major complication of TZD therapy is an increase in the risk of bone fractures. This was first reported in the results of the ADOPT study which was a long-term study to determine the durability of rosiglitazone as a treatment for glycemic control as compared with metformin or glyburide. In this study involving 2511 men and 1840 women, there was an increase in peripheral fractures in women but not men [36••]. After a median treatment of 4.0 years, 60 women (9.3% or 2.74/100 patient years) had at least one fracture. The cumulative incidence of fractures in women at 5 years of treatment was rosiglitazone 15.1%, metformin 7.3%, and glyburide 7.7%. The HR for rosiglitazone-induced fractures was 1.81 and 2.11 relative to metformin or glyburide [36••]. The increase in fractures started after 1 year of treatment. A retrospective analysis of the data from the PROactive study found similar results with an increase in fracture risk in women (5.1% vs. 2.5% or 1.0 vs. 0.5/100 person-years) but not in men (0.6 vs. 0.7/100 person-years) compared with the control population [73].

Two other large randomized, controlled clinical trials reported an increase in fracture risk in TZD-treated patients with T2D. In the RECORD trial which randomized 4447 participants to rosiglitazone or combination metformin and sulfonylurea for a mean of 5.5 years, fracture risk for women was increased by rosiglitazone exposure (2.1/100 person-years compared with the active controls (1.1/100 person-years) but not in men (1.0/100 person-years vs. active controls 0.8/100 person-years) [43••]. In the IRIS trial of 3876 insulin-resistant subjects without diabetes, the 5-year pioglitazone fracture risk was 13.6% compared with the placebo control of 8.8% with a HR of 1.53 [74•]. The fractures were due to a fall in 80%, and 45% were serious and required hospitalization or surgery. The fracture risk from pioglitazone exposure was increased in both men (9.4% vs. active control 5.2%, HR = 1.83) and women (14.9% vs. active control 11.6%, HR = 1.32, *p* = 0.13). The actual fracture rates were 2.3 vs. 1.3/100 person-years for men and 3.7 vs. 2.8/100 person-years for women. The rates in these IRIS participants were higher than in people with diabetes because of the high incidence of falls as these were subjects who had had a previous cerebral ischemic lesion [74•].

In the recently published data from the TOSCA-IT study comparing pioglitazone treatment to an active sulfonylurea therapy in 3028 participants with T2D patients for 57.3 months, there was no significant increases in fractures in pioglitazone exposed female or male participants [42].

Other data on TZD fracture risk come primarily from large medical databases or subanalyses from randomized controlled studies designed for other purposes. In the ACCORD study of intensive vs. ordinary glycemic control, 74% of participants received rosiglitazone and 17% received pioglitazone. During a mean follow-up of 4.8 years, 262 men and 287 women had at least one non-spinal fracture [75•]. There was no increase in fracture risk in men receiving a TZD. Fracture rate in women with 1 to 2 years or > 2 years TZD exposure had HR of 2.32 and 2.01, respectively. Discontinuing TZD use for 1 to 2 years or > 2 years reduced the HR to 0.57 and 0.42 compared with current TZD users [75•].

Database studies confirm the increased risk of fractures in both pre- and postmenopausal TZD-exposed women. Fractures occur starting at 1 year of exposure are usually peripheral and are likely dose- and time-related. Though a few studies find some increase fracture risk in men exposed to TZDs, most do not.

## Pioglitazone and Bladder Cancer

The specter that pioglitazone increases bladder cancer has been an issue for regulators and clinicians for more than a decade. A mismatch in the number of cases of bladder cancer was noted in the PROactive study (14 in the pioglitazone arm and 6 in the placebo arm) [40••]. An epidemiologic study of 1.5 million persons with diabetes followed for 4 years (2002–2006) by the French National Health Insurance Agency reported that pioglitazone-treated males had a statistically significant increase in bladder cancer (HR = 1.22, 95% CI = 1.03–1.43) compared with patients treated with other antidiabetic agents [39]. The risk was increased with a total cumulative dose > 28,000 mg (HR = 1.75) and for exposures > 1 year (HR = 1.34). The French Agency for the Safety of Health Products withdrew pioglitazone from the French market on June 9, 2011, and Germany’s Federal Institute for Drugs and Medical Devices on June 10, 2011, advised physicians not to prescribe pioglitazone until the issue of pioglitazone and bladder cancer was resolved.

A 10-year follow-up study of pioglitazone use and bladder cancer was agreed upon by the Kaiser Permanente Group of California and the US FDA. The study involved 193,099 persons aged 40 years and older in 1997–2002 who were diagnosed with diabetes and managed by the Kaiser health care system and were followed until December 2012 [76••]. Of the cohort, 34,181 (18%) received pioglitazone (mean 2.8 (0.2 to 13.2) years) and 1261 had incident bladder cancer. Ever use of pioglitazone was not associated with the development of bladder cancer (HR = 1.06; 95% CI = 0.89–1.26). In a comparison of 464 bladder cancer patients with 464 matched controls, the adjusted OR for pioglitazone use vs. no use was 1.18 (95% CI = 0.78–1.80) [76••]. Several additional studies support the findings that pioglitazone does not increase bladder cancer [77–79].

## Comparison of Pioglitazone to Rosiglitazone

There have been no randomized, controlled clinical trials comparing the efficacy and safety of rosiglitazone and pioglitazone. The TIDE study which was designed to obtain this information was discontinued after 162 days because of the FDA’s concern about the cardiovascular safety of rosiglitazone and the ensuing public apprehension which would have made recruitment difficult and biased [56]. The only comparative data available are database analyses and meta-analyses.

There are basic studies which do raise the possibility that there may be some differences in the clinical effects of rosiglitazone and pioglitazone. PPARs represent a complicated family of nuclear receptors: PPARα, PPARβδ, and PPARγ [80, 81]. PPARα primarily facilitates fatty acid oxidation and lipid utilization, while PPARγ promotes differentiation of stem cells into adipocytes and facilitates peripheral adipose tissue cells to store rather than release fatty acids. Furthermore, there are three distinct isoforms of PPARγ:PPARγ1 (expressed in almost all tissues), PPARγ2 (expressed mainly in adipose tissue), and PPARγ3 (expressed in macrophages, colon and white adipose tissue). Pioglitazone is a selective human PPARγ1 and a weak human PPARα activator [82]. Chemical proteomics–based analysis profiles show that while there are many common products for pioglitazone and rosiglitazone; there are also many unique products for each [83]. One proven difference between the two TZDs is their effect on human lipid profiles. Rosiglitazone has a less beneficial effect on plasma lipids than pioglitazone: greater increase in VLDL particles; increased LDL particle concentration vs. decrease in LDL particle concentration with pioglitazone; decreased HDL particle concentration and size while pioglitazone increased them [84].

Therefore, it would not be surprising to see differences in the clinical effects of these two TZDs. Unfortunately, as noted above, there are no randomized controlled trials comparing the two. There are database analyses and meta-analyses that conclude that pioglitazone has a greater benefit on cardiovascular outcomes and a lesser side effect profile than rosiglitazone. All these analyses are questionable because they are influenced by selection bias, and the studies that were carried out with rosiglitazone as compared with pioglitazone were suboptimal. For example, PROactive provided much more meaningful data than RECORD. The controversy surrounding rosiglitazone starting in 2007 would be expected to have influenced patient antidiabetic treatment selection and maintenance of treatment in all database analyses. Major unknowns with relevance to side effects and efficacy include the adjustments made in TZD doses and the effects of other drugs, such as diuretics, used to treat fluid retention and heart failure. The development of edema and heart failure appears to be influenced by the underlying degree of vascular disease. In Tables 2 and 3, patients with insulin resistance and prediabetes had very low incidence of edema and heart failure followed by the highest incidence in those who had very significant cardiovascular disease at entry into studies.

## Conclusions

The TZD class of drugs uniquely treats insulin resistance which is the major cause of the increase in T2D and a large component of the increase in atherosclerotic cardiovascular disease. The positive benefits accrued from TZD treatment are summarized in Table 1. The use of these previously extensively prescribed and effective treatments for T2D has been remarkably diminished because of concerns about safety and side effects such as heart failure and increased risk of fractures. Data have been presented to dispel the safety concerns and put the complications into a rational perspective. These drugs are generic, cost is not overly excessive, and they are the most effective pharmacologic agents for treating insulin resistance.
